# Three-dimensional and two-dimensional relationships of gangliogenesis with folliculogenesis in mature mouse ovary: a Golgi–Cox staining approach

**DOI:** 10.1038/s41598-021-84835-0

**Published:** 2021-03-10

**Authors:** Mohammad Ebrahim Asadi Zarch, Alireza Afshar, Farhad Rahmanifar, Mohammad Reza Jafarzadeh Shirazi, Mandana Baghban, Mohammad Dadpasand, Farzad Mohammad Rezazadeh, Arezoo Khoradmehr, Hossein Baharvand, Amin Tamadon

**Affiliations:** 1 Department of Animal Sciences, College of Agriculture, Shiraz University, 71441-65186 Shiraz, Iran; 2 The Persian Gulf Marine Biotechnology Research Center, The Persian Gulf Biomedical Sciences Research Institute, Bushehr University of Medical Sciences, 75146-33196 Bushehr, Iran; 3 Department of Basic Sciences, School of Veterinary Medicine, Shiraz University, Shiraz, Iran; 4 Department of Obstetrics and Gynecology, School of Medicine, Shiraz University of Medical Sciences, Shiraz, Iran; 5 Department of Stem Cells and Developmental Biology, Cell Science Research Center, Royan Institute for Stem Cell Biology and Technology, ACECR, Tehran, Iran; 6 Department of Developmental Biology, University of Science and Culture, Tehran, Iran

**Keywords:** 3-D reconstruction, Microscopy, Cellular neuroscience, Reproductive biology

## Abstract

The present study was set out to investigate two-dimensional (2D) and three-dimensional (3D) evaluations of ovarian nervous network development and the structural relationship between folliculogenesis and gangliogenesis in mouse ovaries. Adult mice ovarian tissue samples were collected from follicular and luteal phases after cardiac perfusion. Ovarian samples were stained by a Golgi–Cox protocol. Following staining, tissues were serially sectioned for imaging. Neural filaments and ganglia were present in the ovaries. In both 2D and 3D studies, an increase in the number and area of ganglia was seen during the follicular growth. The same pattern was also seen in corpora lutea development. However, in some cases such as ratio of ganglia number to follicle area, the ratio of ganglia area to follicular area, 2D findings were different compared with the 3D results. 3D analysis of ovarian gangliogenesis showed the possible direct effect of them on folliculogenesis. Golgi–Cox staining was used in this study for 3D evaluation in non-brain tissue. The results of 3D analysis of the present study showed that, in some cases, the information provided by 2D analysis does not match the reality of ovarian neuronal function. This confirmed the importance of 3D analysis for evaluation of ovarian function.

## Introduction

Follicles are basic units of mammalian ovary. The development of rodent’s follicles begins at neonatal period, the stage at which primordial follicles are formed^1^. Each primordial follicle has an oocyte which is held at first prophase of meiosis and is covered by flattened granulosa cells layer^2^. After female maturation the estrous cycle starts. Through this cycle, primary, secondary, antral and preovulatory follicles develop from primordial follicles^3^. At this stage, most of the antral follicles undergo atretic degeneration and a few of them, under stimulation of follicle-stimulating hormone (FSH) and luteinizing hormone (LH), become preovulatory follicles^2,4^. After that, due to follicle response to LH hormone, the follicle ovulates. The remaining cell transforms and forms corpus luteum^4^. The ovarian cycle in mouse strains is called estrous cycle. This cycle includes four stages: proestrus and estrus as follicular phase and metestrus and diestrus as luteal phase^5^. Ovulation and corpus luteum formation occurs in estrus stage. Presence of corpus luteum is vital for progesterone secretion ^6,7^. So, due to lack of pregnancy, corpus luteum undergoes degeneration and progesterone secretion reduces at proestrus stage. Hence, next estrous cycle starts^8^.

The mammalian’s ovary is regulated by hormonal factors and direct neuron effects^9^. Several studies have demonstrated that in mouse strains there are distinct populations of neurons, both internal and external neurons. It has been shown that the chemical phenotypes of ovarian neurons of some mouse strains are sympathetic, similar to primates^10^. Due to results of previous studies, it is now well established that ovarian neurons are derived from neural crest cells, which form with complete ovarian maturation and subsequent reproductive functions^11^. Noradrenergic nerves are expressed in the ovary near birth^12^. It has been conclusively shown that the total number of neurons in puberty increases and then decreases^12^. External nervous system of mouse ovary has many roles. Several studies have shown its role in developmental process, cyclic stages, pregnancy, and aging process^13–15^. These nerves and ganglia are responsible for ovarian estradiol secretion^15^. The number of internal neurons in neonatal ovaries was lower than that of adult ovaries, some of which form ganglia and networks^16^.

Some neurotransmitters such as neurotrophins have an important role in follicle growth. For example, reduction of brain-derived neurotrophic factor (BDNF) and neurotrophin-4 (NT-4) can cause folliculogenesis disorder^17^. Also, these nerves and ganglia can take part in pathological conditions such as polycystic ovary syndrome (PCOS)^18^. Based on the previous studies, hypothetically assumed that the ganglia may have an important role in mice ovary function. In spite of the fact that many researchers have utilized the two-dimensional (2D) methods for evaluation of ovarian nervous system, so far no three-dimensional (3D) analysis research has been performed on ovarian ganglia using image processing techniques and non-immunostaining methods. The purpose of the present study was to perform three-dimensional evaluation of ovarian ganglia network development and their structural relationship with folliculogenesis in the mice ovary. Golgi–Cox staining was performed in the ovary for imaging of ganglia.

## Results

### Ganglia and ovarian structures in Golgi–Cox staining

**Figure 1:**
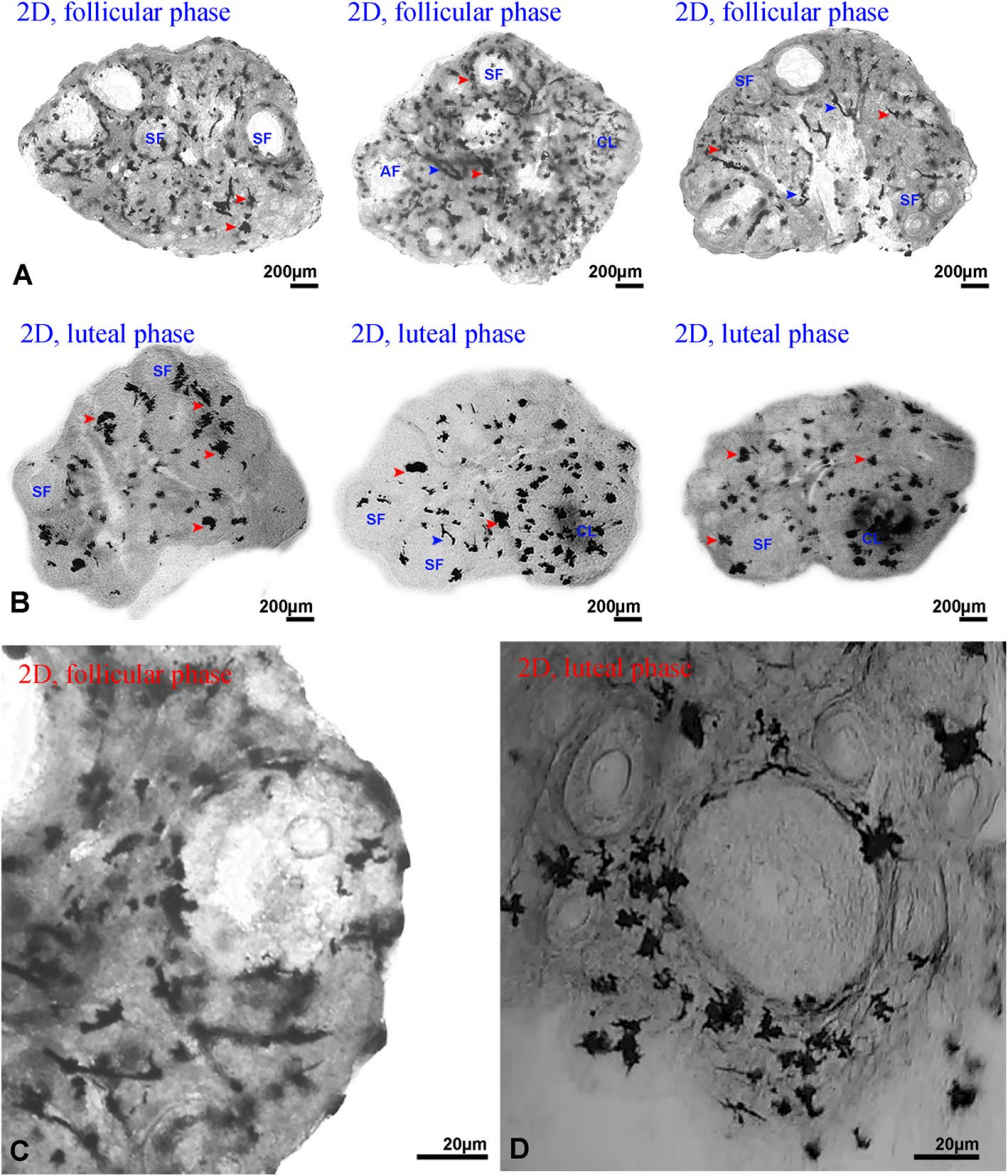
Mouse ovarian sections were stained with Golgi–Cox method. (**A**) Two-dimensional follicular phase ovary. (**C**) Distribution of ganglia around follicular phase ovarian follicles. (**D**) Distribution of ganglia around luteal phase ovarian follicles. The ganglia (red arrow head), and neural filaments (blue arrow head) are stained black. Secondary follicles (SF), Antral follicles (AF) and corpus luteum (CL) are also seen (all images are gray scale format).

**Figure 2:**
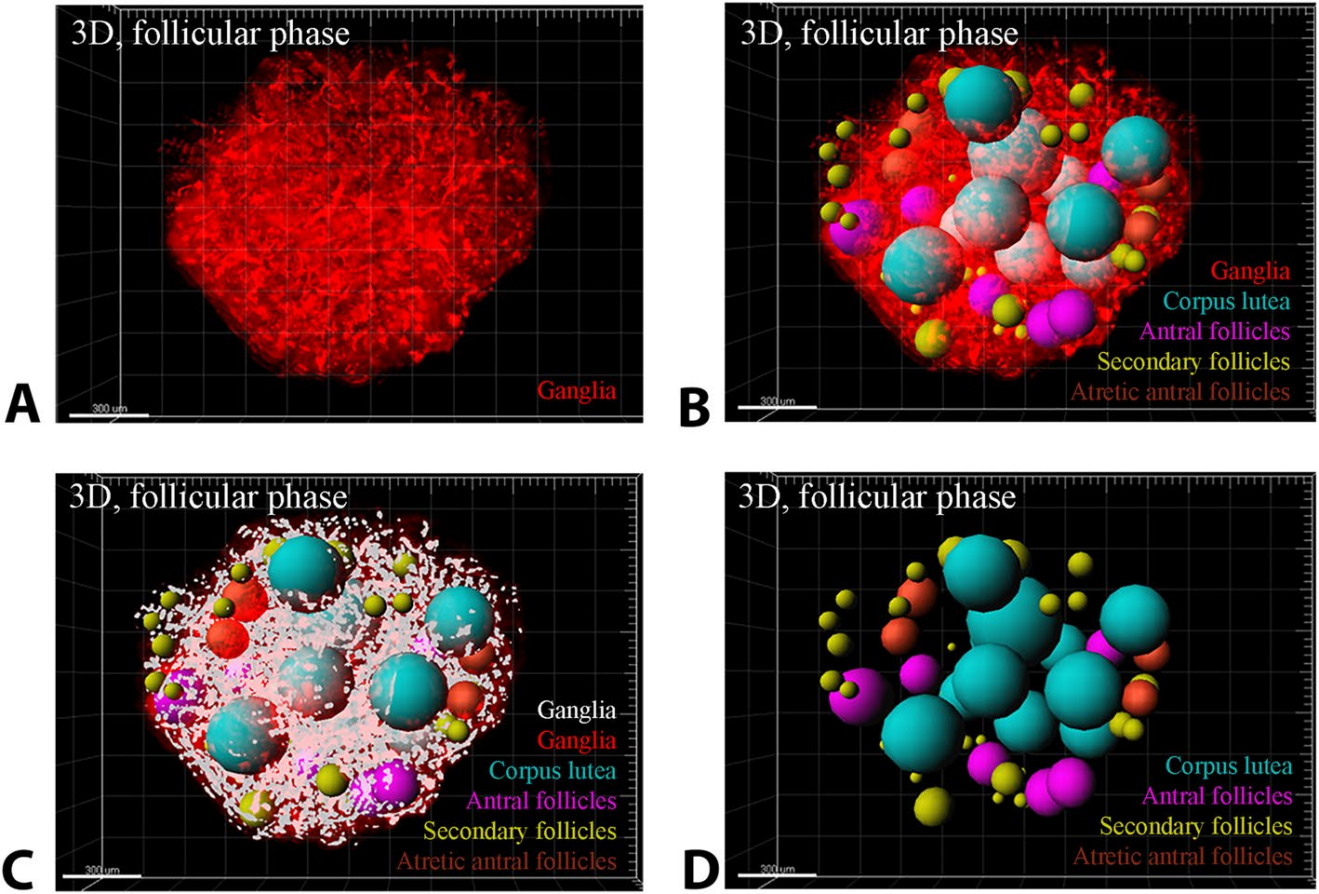
Three-dimensional (3D) reconstruction and segmentation of mice ovary structures after Golgi–Cox staining for detection of ovarian structures relationship with ganglia networks. (**A**) Whole tissue imaging and 3D reconstruction of mice ovary after Golgi–Cox staining (red color represent the ganglia); (**B**) ovarian follicles and corpora lutea segmentations in the whole tissue 3D reconstructed ovary. The blue spots represent Antral follicles, yellow spots represent secondary follicles, purple spots represent corpus luteum and brown spots represent atretic antral follicles. (**C**) Ovarian follicles, corpora lutea and ganglia segmentations. Ganglia are shown with white cells. (**D**) Ovarian follicles and corpora lutea segmentations. All scale bars in four images are 300 µm. All images are processed and drawn by Imaris software.

In the present study, Golgi–Cox staining was used for the first time to identify the ganglia network of mouse ovaries. Also, with the help of this method of staining and serial cryo-sectioning technique and three-dimensional reconstruction of ovarian slices, the parameters of gangliogenesis relationship with ovarian structures were compared. Briefly, in 2D images, the ganglia structures were stained black and the ovarian tissue was stained brown (Fig. 1). Conversely, in 3D images, the ganglia structures were recolored red and the ovarian tissue color was changed to transparent for 3D reconstruction (Fig. 2, Video S1).

**Figure 3:**
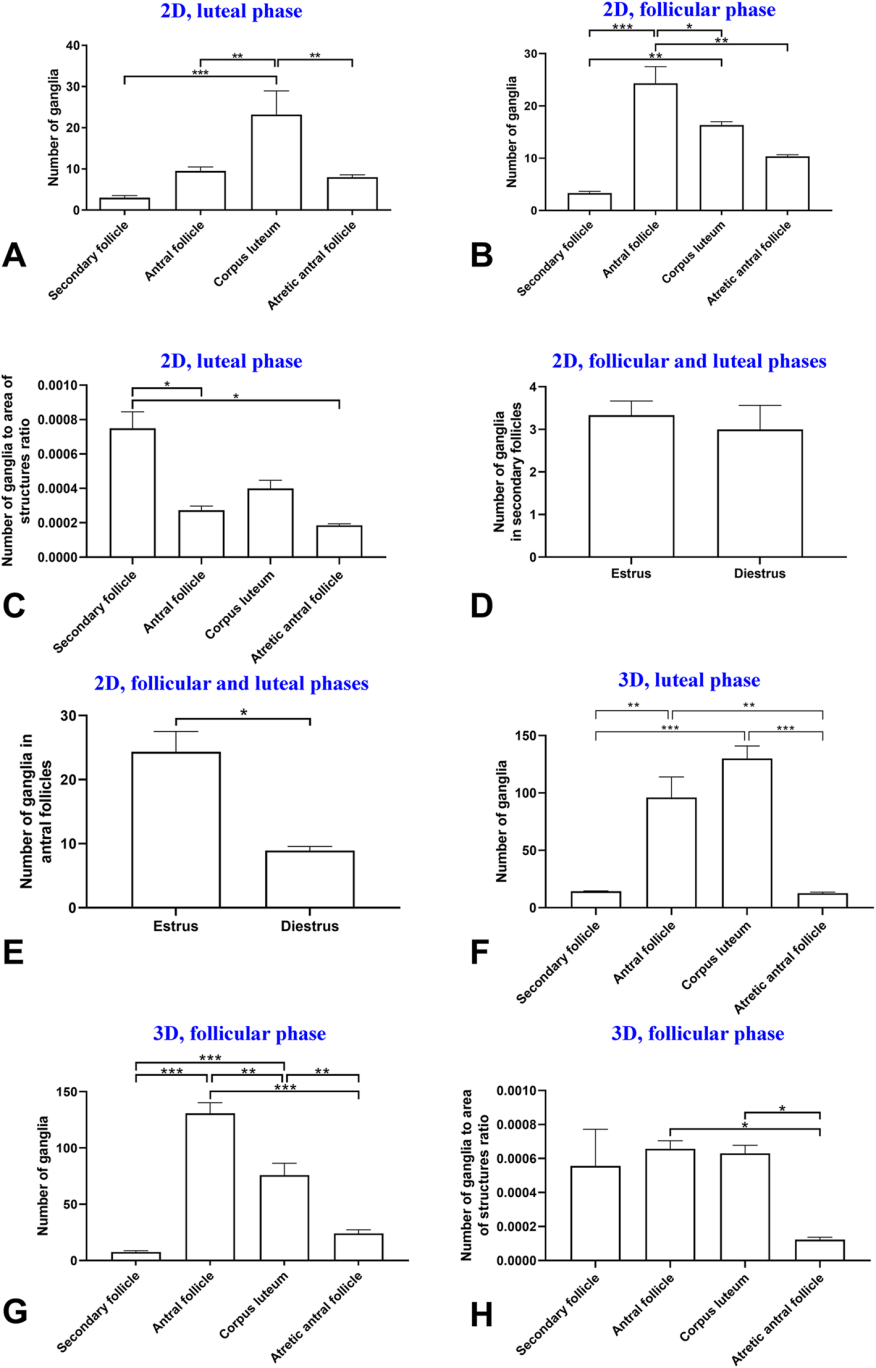
Comparisons of means and standard errors of variables in the three-dimensional (3D) and two-dimensional (2D) studies show the relationship of follicular growth and increase in ganglia number in the mouse ovary. Number of ganglia in luteal phase (**A**) and follicular phase (**B**) phases in follicles and corpora lutea in the 2D study. (**C**) Number of ganglia to area of structures ratio in luteal phase in the 2D study. (**D**) Number of ganglia in secondary follicles in follicular and luteal phase ovary in the 2D study. (**E**) Number of ganglia in antral follicles in follicular and luteal phase ovaries in the 2D study. (**F**) Number of ganglia in follicles and corpus luteum in the 3D study. (**G**) Number of ganglia to area of structures ratio in the 3D study (*p < 0.05, **p < 0.01, ***p < 0.001).

In addition, the neural network was detectable in this color in black, but with continuous filament structures in 2D (Fig. 1A) and 3D images (Fig. 2A). The ganglia were also recognizable as a network of tree dendrites from a cell body between the theca and granulosa cell layers around all types of follicles (Fig. 1B,C). In corpora lutea, these ganglia were scattered throughout the corpus luteum structure (Fig. 1A). The size, the shape and the number of branches were structurally different between ganglia but all of them were multipolar (Fig. 1B,C). The ganglia of the follicular phase ovaries (Fig. 1A,B) and the luteal phase ovaries (Fig. 1C,D) were measured and compared. The number of the ganglia at luteal phase ovary seems to be lower than the follicular phase ovary (Fig. 1A,D).

After reconstruction of ovarian structures by 3D method, scattering of ganglia between follicles and corpus luteum was observed (Fig. 2B). The segmentation of the ganglia after the segmentation of the follicular structures made it possible to image the spatial relationship between both ganglia networks and reproductive structures (Fig. 2C). The spot algorithm for measuring follicles and corpora lutea completely segmented the ovarian structures (Fig. 2B,D). The cell algorithm also segmented the network structures of the ganglia (Fig. 2C). Ganglia and neurons were observed in all parts of the ovarian tissue. Nerve tissue density especially neural filaments was higher in the medulla of ovaries than the cortex.

### Follicular growth and increase of ganglia number

In the 2D study, the total number of ganglia increased during follicular growth (*p* < 0.05; Fig. 3A,B). In contrast with luteal phase ovary, in follicular phase ovary, the total number of ganglia in the antral follicles was higher than in the secondary follicles and atretic antral follicles (*p* < 0.001 and *p* = 0.001, respectively; Fig. 3A,B). Investigating changes in the number of ganglia relative to increasing follicle area in the 2D study, it was observed that the ratio of ganglia number to follicle area in secondary follicles was higher than antral follicles and atretic antral follicles (*p* = 0.023 and *p* = 0.022, respectively, Fig. 3C). However, the number of ganglia in secondary follicles in the follicular and luteal phases was not significantly different (*p* > 0.05, Fig. 3D), but the number of ganglia in antral follicles in follicular phase was higher than luteal phase (*p* = 0.03, Fig. 3E).

On the other hand, in the 3D study, the total number of ganglia increased during follicular growth, as well as 2D study (*p* < 0.05; Fig. 3F). Indeed, the total number of ganglia in the antral follicles was higher than the secondary and atretic antral follicles in both luteal and follicular phase ovaries (*p* < 0.01 and *p* < 0.001, respectively; Fig. 3F,G). Furthermore, the ratio of ganglia number to follicle area in the secondary follicles and the antral follicles was not different, which was in contrast with the 2D study findings (*p* > 0.05; Fig. 3H). In addition, this ratio in the antral follicles was higher than atretic follicles, unlike the 2D study analysis (*p* = 0.026; Fig. 3H).

### Follicular growth and increase of ganglia area

**Figure 4:**
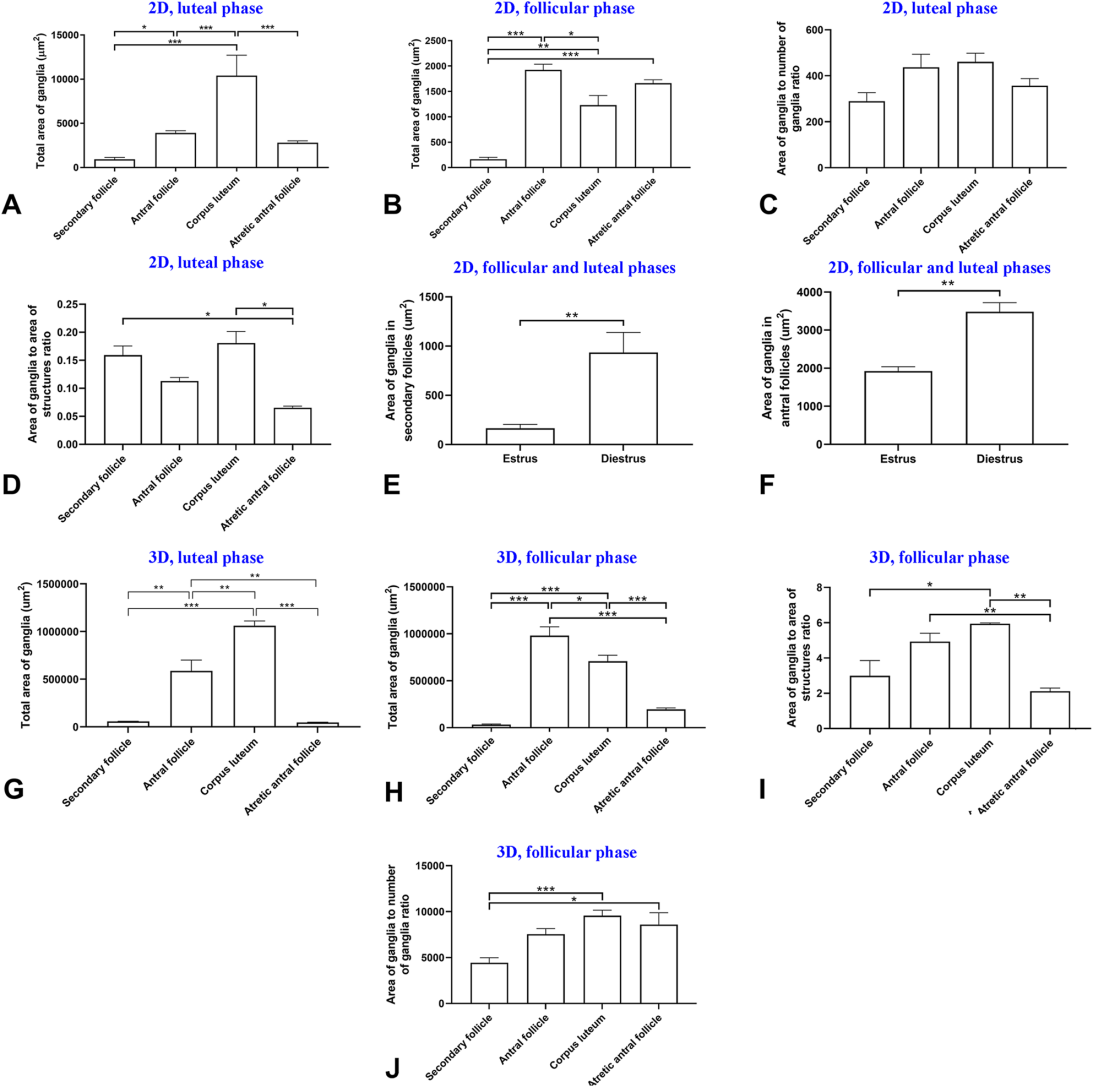
Comparisons of means and standard errors of variables in the three-dimensional (3D) and two-dimensional (2D) studies to show the relationship of follicular growth and increase in ganglia area in the mouse ovary. Area of ganglia in luteal phase (**A**) and follicular phase (**B**) phases in follicles and corpora lutea in the 2D study. (**C**) Area of ganglia to number of ganglia ratio in luteal phase in the 2D study. (**D**) Area of ganglia to area of structures ratio in luteal phase in the 2D study. (**E**) Area of ganglia in the secondary follicles in follicular and luteal phases in the 2D study. (**F**) Area of ganglia in the antral follicles in follicular and luteal phases in the 2D study. (**G**) Area of ganglia in follicles and corpora lutea in the 3D study. (**H**) Area of ganglia to area of structures ratio in the 3D study. (**I**) Area of ganglia to number of ganglia ratio in the 3D study (*p < 0.05, **p < 0.01, ***p < 0.001).

In 2D study, the total area of ganglia increased during follicular growth (*p* < 0.05; Fig. 4A,B). Total area of ganglia in the antral follicles was higher than the secondary follicles in both luteal and follicular phase ovaries (*p* = 0.48 and *p* < 0.001, respectively; Fig. 4A,B). In addition, the ratio of ganglia area to ganglia number between the antral follicles and the secondary follicles was not different (*p* > 0.05, Fig. 4C). However, the ratio of ganglia area to area of structures in the secondary follicles was higher than the antral follicles (*p* > 0.05, Fig. 4D). In addition, the ratio of ganglia area to area of structures in the secondary follicles was higher than the atretic antral follicles (*p* = 0.034, Fig. 4D). Furthermore, the area of ganglia in the secondary follicles in luteal phase ovary was higher than the follicular phase ovary (*p* = 0.001, Fig. 4E). Also, the area of ganglia in the antral follicles in luteal phase ovary was higher than the follicular phase ovary (*p* = 0.006, Fig. 4F).

In the 2D study, there were positive correlations between increase in ganglia area and increase in ganglia number; increase in ganglia area and increase in ovarian structures’ area; and increase in ganglia number and increase in ovarian structures’ area (*p* = 0.0001, Table 1). In addition, in the secondary follicles, there were positive correlations between increase in ganglia area and increase in ganglia number; increase in ganglia area and increase in secondary follicles’ area and increase in ganglia number and increase in secondary follicles’ area (*p* = 0.0001, Table 1). Moreover, in the antral follicles and the atretic antral follicles, there was also positive correlation between these three groups (*p* = 0.0001, Table 1).

**Table 1:** Correlation coefficient of follicular area and ganglia development including ganglia number and area in different follicular phases and corpus luteum in two-dimensional ovary analysis.

Structures	Ganglia area-ganglia number		Ganglia area-structure area		Ganglia number-structure area	
	Correlation coefficient	*p*-value	Correlation coefficient	*p*-value	Correlation coefficient	*p*-value
Ovarian structures	0.971	0.0001	0.924	0.0001	0.925	0.0001
Secondary follicle	0.880	0.0001	0.920	0.0001	0.882	0.0001
Antral follicle	0.956	0.0001	0.904	0.0001	0.983	0.0001
Corpus luteum	0.955	0.003	0.950	0.004	0.932	0.007
Atretic antral follicle	0.993	0.0001	0.976	0.0001	0.993	0.0001

In the 3D study of luteal phase ovaries, the total area of ganglia in antral the follicles was higher than the secondary and atretic antral follicles (*p* < 0.01; Fig. 4G). On the other hand, in the 3D study of follicular phase ovaries, the total area of ganglia increased during follicular development (*p* < 0.05; Fig. 4H). Area of ganglia in the antral follicles was higher than the secondary follicles, as well as the 2D study (*p* < 0.001, Fig. 4H). In contrast with the 2D study, the ratio of ganglia area to follicular area in the antral follicles was not different with the secondary follicles (*p* > 0.05, Fig. 4I). Additionally, the ratio of ganglia area to follicular area in the antral follicles was higher than the atretic antral follicles, which it was not seen in 2D study (*p* = 0.009, Fig. 4I). However, the ratio of ganglia area to ganglia number in the atretic antral follicles was higher than the secondary follicles (*p* = 0.017, Fig. 4J).

In the 3D study, the same as the 2D study, there were positive correlations between increase in ganglia area and increase in ganglia number; increase in ganglia area and increase in ovarian structures’ area; and increase in ganglia number and increase in ovarian structures’ area (*p* = 0.0001, Table 2). In addition, in the secondary follicles as well as the 2D study, there were positive correlations between increase in ganglia area and increase in ganglia number; increase in ganglia area and increase in secondary follicles’ area; and increase in ganglia number and increase in secondary follicles’ area (*p* < 0.05, Table 2). In contrast with the 2D study in the antral follicles, there were no correlations between increase in ganglia area and increase in ganglia number; increase in ganglia area and increase in antral follicles’ area; and increase in ganglia number and increase in antral follicles’ area (*p* > 0.05, Table 2). In addition, in contrast with the 2D study in the atretic antral follicles there were no correlations between increase in ganglia area and increase in ganglia number; increase in ganglia area and increase in atretic antral follicles’ area; and increase in ganglia number and increase in atretic antral follicles’ area (*p* > 0.05, Table 2).

**Table 2:** Correlation coefficient of follicular area and ganglia development including ganglia number and area in different follicular phases and corpus luteum in three-dimensional ovary analysis.

Structures	Ganglia area-ganglia number		Ganglia area-structure area		Ganglia number-structure area	
	Correlation coefficient	*p*-value	Correlation coefficient	*p*-value	Correlation coefficient	*p*-value
Ovarian structures	0.985	0.0001	0.978	0.0001	0.979	0.0001
Secondary follicle	0.966	0.008	0.948	0.014	0.878	0.05
Antral follicle	0.532	0.468	0.566	0.434	0.429	0.571
Corpus luteum	0.927	0.023	0.997	0.0001	0.918	0.028
Atretic antral follicle	0.540	0.460	0.667	0.333	0.857	0.143

### Corpus luteum development and increase of ganglia parameters

In the 2D study, the total area and number of ganglia increased during corpus luteum development (Figs. 2A,B,3A,B). Specifically, the total number of ganglia in the corpora lutea was higher than the antral, and secondary and atretic antral follicles in luteal phase ovary (*p* < 0.001, *p* = 0.002 and *p* < 0.002, respectively Fig. 3A). Additionally, the total number of ganglia in the antral follicles was higher than the corpus luteum in follicular phase ovary (*p* = 0.036, Fig. 3B). In both luteal and follicular phases, number of ganglia in corpora lutea was higher than the secondary follicles (*p* < 0.001 and *p* = 0.002, respectively; Fig. 3A,B).

**Figure 5:**
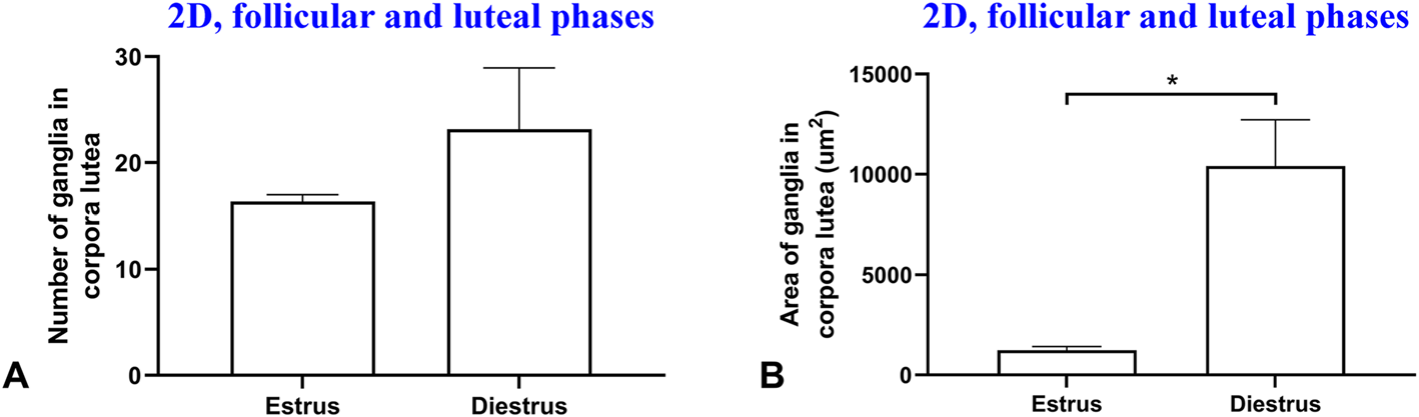
Comparisons of means and standard errors of number of ganglia in the two-dimensional studies to show the relationship of corpora lutea function and increase in ganglia number (**A**) and area (**B**) in the mouse ovary.

In 2D study, the ratio of ganglia number to area of structures and also the ratio of ganglia area to ganglia number in the corpora lutea and the antral follicles was not different (*p* > 0.05, Figs. 3C,4C). Also, the ratio of ganglia area to area of structures in the corpora lutea was more than the atretic antral follicles (*p* = 0.025, Fig. 4D). Moreover, number of ganglia in the corpora lutea in luteal and follicular phases was not different (*p* > 0.05, Fig. 5A). In contrast, the area of ganglia in the corpora lutea in luteal phase ovary was higher than follicular phase ovary (*p* = 0.01, Fig. 5B).

In the 2D analysis, there were positive correlations between area of ganglia and number of ganglia in the corpora lutea (*p* = 0.003, Table 1). Also, positive correlations between area of ganglia and area of structure and number of ganglia and area of structure in the corpora lutea were observed (*p* = 0.004 and *p* = 0.007, respectively; Table 1).

In 3D analysis of luteal phase ovaries, the total number of ganglia in the corpora lutea was higher than the secondary and atretic antral follicles (*p* < 0.001, Fig. 3F). In addition, in the 3D study of follicular phase ovaries, total number of ganglia in the antral follicles was higher than the corpora lutea in follicular phase (*p* = 0.001, Fig. 3G) The number of ganglia in corpora lutea was higher than the secondary and the atretic antral follicles in follicular phase ovary (*p* < 0.001 and *p* = 0.002, respectively, Fig. 3G), same as the 2D analysis of luteal phase ovary. The 3D study results of luteal phase ovaries were almost similar to the 2D study. In the 3D analysis of luteal phase ovaries, the total area of ganglia in the corpora lutea was higher than the secondary, antral and atretic antral follicles (*p* < 0.001, *p* = 0.003 and *p* < 0.001, respectively; Fig. 4G). In both the 2D and 3D study of follicular phase ovaries, total area of ganglia in the antral follicles was higher than the corpora lutea (*p* = 0.013 and *p* = 0.025, respectively, Fig. 4B,H) and higher than secondary follicle (*p* < 0.001, Fig. 4H). Moreover, the total number and total area of ganglia in the corpora lutea were higher than the atretic antral follicles (*p* = 0.002 and *p* < 0.001, respectively, Figs. 3F,4H). Furthermore, the ratio of ganglia area to ganglia number between the corpora lutea and the antral follicles was not different (*p* > 0.05, Fig. 4J). In contrast, the ratio of ganglia area to ganglia number in the corpora lutea was higher than the secondary follicles (*p* = 0.004, Fig. 4J). The area of ganglia to area of structures ratio in the corpora lutea was higher than the secondary and the atretic antral follicles (*p* = 0.006 and *p* = 0.001, Fig. 4I). In contrast, this ratio between the corpora lutea and the antral follicles was not different (*p* > 0.05, Fig. 4I).

In the 3D analysis, there were positive correlations between area of ganglia and number of ganglia in the corpora lutea (*p* = 0.02, Table 2), area of ganglia and area of structure (*p* < 0.001, Table 2) and number of ganglia and area of structure (*p* = 0.02, Table 2).

## Discussion

In the present study the gangliogenesis during folliculogenesis and their relationship was shown in both 2D and 3D evaluation of mice ovaries. We found that during folliculogenesis the area and number of ganglia in the follicular wall increased. Also, the 3D study results revealed that although the number of ganglia did not increase by development of the secondary follicles to the antral follicles, proportionally, considering the increase of volume and area of follicles during folliculogenesis, the area of ganglia increased from the secondary follicles to the antral follicles. As a result, proliferation and hypertrophy of ganglia were observed during folliculogenesis in mice ovary. These phenomena were observed in both follicular and luteal phases. Besides, during follicular development, their function increased, too^19^. Therefore, due to their enhanced function, secretion activity increased, subsequently^19^. Furthermore, in a 3D evaluation of mouse ovary it has been shown that angiogenesis happened during folliculogenesis^20^. On the other hand, previous studies have shown that there is some relationship between internal nervous network and vascular system in ovary functions and both of them take part in ovarian secretion^18,21–23^. They indicated that vessels and nerves have an important role in folliculogenesis and ovulation^18,21–23^. Consistent with our findings on the relationship between ovarian function and gangliogenesis, the same findings about other neuronal cells in mice^10,24^, monkeys and humans^9,25^ demonstrated that ovarian internal neuronal filaments increased in sexual maturation. Furthermore, distribution, morphology, and chemical phenotype of ovarian intrinsic nervous system in guinea pigs increase in adult animals compared with neonates^16^. Comparing previous findings with our results, it can be speculated that follicular function has a positive relationship with gangliogenesis, intrinsic neuronal network and vasculogenesis. Another result of current study showed that the estrous ovary had higher number of ganglia of antral follicles than luteal phase ovary, however, their area was higher in luteal phase than follicular phase ovary. These results suggest that during follicular phase there is ganglia proliferation around the antral follicles. In addition, it is further conducted that at the luteal phase the ganglia undergo hypertrophy and the area of the ganglia increase.

On the other hand, data of the current 3D study demonstrated that the number of ganglia to area of structures ratio in the secondary and antral follicles and corpora lutea were not different. As the area of the follicles enlarges, which indicates an increase in the number of granulosa and theca cells vascularization^26^, the number of ganglia has increased to such an extent that the ratio of the ganglia number to the area of the follicles and corpora lutea remains constant. This indicates a constant ovarian structure volume-dependent gangliogenesis. The number of ganglia to area of structures ratio remains high during follicular development. Due to the increase in ovarian follicular metabolism^27^ and in hormonal activity^28^, to ensure the interaction of ovarian structures and ganglia remain constant, the area and volume of ganglia, as well as ganglia number increase.

During follicular development, some follicles undergoes atresia^29^. The specific criteria for follicles undergo atresia, which it called atretic follicles, is pyknotic nuclei^30^. However, other criteria for atretic follicles recognition is follicular deformity and they don’t have rounded shape^29^. As the Golgi–Cox staining only stain the cells of nervous system^31^, the pyknotic nuclei in granulosa cells around the follicles are not recognizable. In the present study, the atretic antral follicles were distinguished by their shapes’ deformity and non-rounded shape from antral follicles. Totally, in the follicular phase ovary, the total number and area of ganglia in atretic antral follicles were lower than antral follicles in both 2D and 3D studies. The follicles in follicular phase became almost mature and on the other hand the rest of the follicles undergo atresia^29^. This showed that the as the atretic follicles undergoes degeneration and their cells reduced, their ganglia also reduced in number and area. However, in the luteal phase ovary, there was no significant difference between antral follicles and atretic antral follicles which it could be because of the less maturation of antral follicles in luteal phase ovary^29,32^.

The corpora lutea has the highest value of ganglion area and number compared with the other ovarian structures in luteal phase. This result was observed at both 2D and 3D analysis of luteal phase ovaries. Along with these results, the study of mice estrous cycle showed that progesterone secretion of the corpora lutea increased in luteal phase^33^. In early luteal phase, area of luteal tissue and its function increased, which is known as luteogenesis phase^34^. In addition, results from rat ovary showed that the corpora lutea formed at early luteal and progesterone secretion started^35^. These results indicated that corpora lutea in luteal phase has a high function and produces progesterone. In order to reach this function, corpora lutea needed more secretory cells. On the other hand, ganglia of the corpora lutea decreased during its atretic degradation. As the ovary was in follicular phase, the results showed that the antral follicle has higher value of ganglia’s area and number than the corpora lutea. Due to lack of pregnancy, the corpora lutea begin degradation and at the follicular phase are no longer observed^8,36^. Therefore, it can be resulted that during degradation of the corpora lutea, its ganglia undergo degradation too. The 3D results confirmed our 2D findings and showed degradation of ganglia during degradation of the corpora lutea. The results of previous studies demonstrated that the corpora lutea has higher vessels and angiogenesis^37^, which shows its higher activity rather than the other ovarian structures. Due to the fact that the nervous system of ovary has a role in regulation of mammalian ovary function, steroidogenesis and ovulation^38^ and ganglia may have roles in progesterone secretion, also, the higher number of ganglia in corpora lutea at luteal phase was seen in comparison with regressing corpora lutea in the follicular phase.

To the best of our knowledge, this is the first time that 3D morphological parameters of ganglia in the ovarian follicles and the corpora lutea of mice were determined. In contrast with the 2D study, in the 3D study, the number of ganglia to area of structures ratio in the secondary and the antral follicles was not different. This finding showed that during follicular growth, due to preserving the function of follicles, this ratio always remained constant, which cannot be seen by the limitations of the 2D analysis. In some other findings of our study, the results of the 2D study were different from the 3D study and the 2D analysis could not give us a proper aspect of the view of reality compared to the 3D analysis. In line with our findings, even results of the 2D and 3D ultrasound techniques in ovary showed that the 2D analysis could not detect all data of ovarian structures and the 3D analysis was realistic^39^. In addition, in a histomorphological evaluation of rodents’ brain, the 3D analysis was more accurate in analyzing all parts of brain rather than the 2D analysis.

In the present study, although the cost of immunohistochemistry method for whole tissue imaging of neuronal networks of brain^40^ and different organs makes this approach unconventional, Golgi–Cox method was performed for nervous tissue labeling and 3D imaging. Golgi staining is one of the oldest staining methods for nervous system imaging and was developed by Camillo Golgi at 1873^41^. This method has been shown to be effective due to neuronal morphology imaging such as dendritic and axonal arborization and spines detection^42^. There are three methods of Golgi staining: Golgi–Cox, Rapid Golgi and Golgi–Kopsch^43,44^. While all of these methods have some different advantages and disadvantages, Golgi–Cox method is better than the others for dendritic trees analysis due to less background density^44^. In this method neurons, especially dendritic trees stain clearly without noise^44^. Four types of ganglia exist in ovary of young and postnatal adult rats, including mesovarial, Hilar, medullary, and cortical ganglia^24^. In the present study, Golgi–Cox method based on the previous study “Golgi–Cox staining step by step”^45^ with slight modification was performed.

In conclusion, the present study demonstrates the positive relationship of gangliogenesis during folliculogenesis in mouse ovary. Furthermore, gangliogenesis has been shown in corpus luteum development. Ovarian ganglia as an independent part of ovarian nervous system is likely to have an important role in folliculogenesis and luteogenesis. Furthermore, 3D analysis instead of conventional 2D approach, in addition to Golgi–Cox staining of ovary can be used for study of this physiological phenomena in ovary.

## Methods

### Animals

The ovaries of 12 non-pregnant adult BALB/c mice were used in the present experimental study. The mice weighed 35 ± 2 g and were 49 days old. Mice had free access to food and water and were kept in laboratory cages in standard conditions at 22 °C and 12 h light/dark cycle. Complete ovarian sample was used for imaging and statistical analysis.

Briefly, after the mice were anesthetized, 0.9% NaCl solution was perfused in left ventricle of the mouse hearts. Then the ovaries were removed from the mice, and the pattern of nervous system distribution with intra-ovarian origin and their relation to folliculogenesis was determined by cross-sectional imaging and image analysis of the ovarian tissues. According to the protocol, the initial experiments were performed so as to repeat the different times of the samples in Golgi–Cox solution, showing the stability of this staining and the most appropriate result.

### Estrous cycle evaluation

The vaginal smear test was performed to detect estrous cycle. Using 100 µl of physiological salt solution, aspiration of vaginal canal was done. Aspiration was considered about 4 times on each mouse. The contents of the sampler’s head were then drained onto the slide and coated with cover slips. Finally, based on previous study^5,46^, using light microscopy estrous cycle phases for mouse was determined. Briefly, predominant cornified squamous epithelial cells observation indicated follicular phase. However, at luteal phase, the cornified squamous epithelial cells was not (or rarely) observed. At luteal phase, the most predominant cells were leukocytes.

### Cardiac perfusion and ovary collection

The mice were anesthetized by chloroform-impregnated cotton. Then dissecting the heart, the right atrium was cut and 0.9% saline solution was injected into the left ventricle. The saline solution slowly entered the circulatory tract. The perfusion was then performed with a syringe containing 10 mL of 4% formalin until the tissues became pale. After this, the ovaries were removed.

### Golgi–Cox staining

Staining was done by a modified Golgi–Cox technique as described by Zaqout et al.^45^. This modified method includes three main solutions.

- Solution A: 5% (w/v) solution of potassium dichromate (K_2_Cr_2_O_7_; UNI-CHEM, Serbia).
- Solution B: 5% (w/v) solution of mercuric chloride (HgCl_2_; UNI-CHEM, Serbia).
- Solution C: 5% (w/v) solution of potassium chromate (K_2_CrO_4_; UNI-CHEM, Serbia).

All three solutions were kept in glass bottles at room temperature in the dark. In this condition, these solutions can be used for a long time. For preparation of impregnation solution 25 mL of solution A was slowly mixed with 25 mL of solution B. 20 mL of solution C was carefully added to previous mixture. In the final step, 50 mL of dd-H_2_O was added to the mixture of all three solutions. Final solution was covered with aluminum foil and was kept at room temperature in completely dark condition for 48 h. After that, the reddish-yellow precipitation was formed. The supernatant solution was gently collected by glass pipet (avoiding the reddish-yellow precipitant in supernatant collection). The ovary tissue was transferred into glass bottle containing Golgi–Cox solution. After 24 h, the tissue was transferred into fresh Golgi–Cox solution. The glass bottle was kept in the dark at room temperature for 14 days.

### Ovary cryo-sectioning

Firstly, cryoprotectant solution was prepared. For cryoprotectant preparation, 30 g of sucrose was dissolved in 100 mL ddH_2_O (30% sucrose solution). After tissue impregnation in Golgi–Cox solution, tissues were transferred in cryoprotectant solution. The samples were kept in this solution at 4 °C in dark for 24 h. After that, cryoprotectant solution was refreshed and the samples were kept in new solution in the same conditions for 5 days. Then samples were washed with ddH_2_O and fixed on cryo-holder by cryo-glue. Finally, the samples were transversely sectioned on a cryo-holder at a temperature of − 25 °C and a thickness of 30 μm. The serial sections were transferred onto gelatin coated slides for developing stage on staining.

### Developing step of Golgi–Cox staining

The developing step of Golgi–Cox staining was performed. In detail, slides were kept in ddH_2_O twice for 2 min each time. For dehydration of sections, slides were placed in 50% ethanol for 5 min. After that, sections were transferred into ammonia solution (3:1 ammonia to ddH_2_O) for 6 min. Then slides were placed in ddH_2_O twice, each for 2 min. At the next step, samples were kept in 5% sodium thiosulfate solution for 10 min in dark condition. After repeating the ddH_2_O step twice (1 min each), samples were dehydrated with ascending percentage of ethanol (70%, 95%, and 100% ethanol each for 5 min). After dehydration step, tissues were placed in xylol for 10–15 min until tissues were completely cleared. In the final step, slides were mounted by Entellan glue. Sections were kept in the dark until imaging.

### Ovarian tissue imaging

An optical microscope (Nikon, E200, Japan) with a 40 × magnification and a Dino Capture camera (AnMo Electronics Corporation, New Taipei City, Taiwan) were used. Whole ovarian serial sections images were captured for each ovary. The images were provided in 2600 × 1950 pixels with TIFF format. The numbers and area of follicles, corpus lutea, ganglia and neuronal filaments were calculated. The criteria for assortment of follicles and corpus luteum in 2D analysis was described in Azarnia et al.^47^ and for 3D analysis it was described in Feng et al.^48^. In addition, the assortment of the atretic antral follicles were described^29^.

### 2D analysis of ovarian follicular and neuronal structures

ImageJ software used for 2D image analysis of ovarian structures. Firstly, imported images converted to 8-bit images using “Image type” option in “Image” panel. Then scale of all images was set using “Set scale” option in “Analyze” panel. In order to analyze single follicle, the area of each follicle containing its surrounded ganglia and a few neuronal filaments was manually cropped. For this purpose, the border of each follicle with its ganglia were selected by “Oval” tool in main menu of ImageJ software (Figure S1A). After that, the selected area was duplicated and analysis was continued. The specified structures (ganglia and neural filaments) were measured by “threshold” algorithm. The size of threshold field was adjusted by dragging the threshold border, manually. By adjusting the signal threshold in the software, the shape of the ganglia and the neuronal filaments around the follicles were detected. Using “Analyze particles” in analyze panel, the number and area of particles were measured (Figure S1B). In this stage, filaments in a few crops were deleted manually using “delete” option in “ROI manager”. Finally, data were measured by measure option in ROI manager and data were saved as an excel file with CSV format (Figure S2A).

### 3D analysis of follicular and neuronal structures

First, using ImageJ software, serial images were combined as a TIFF series image using “Images to Stack” tool. The TIFF series image was saved as stack with TIFF format. Then, using Imaris software (V 7.4.2, ImarisX64, Bitplane AG), 3D reconstruction was performed. Specifically, after serial TIFF image was imported, the dimension of image was corrected in z-stack according to thickness of tissue slices using “Image properties” in “Edit” panel. Firstly, the whole image was reconstructed. In order to reconstruct follicles and corpora lutea, “Spot” algorithm was used in “Surpass” panel. This procedure was done manually in order to cover follicle area and diameter. Three “Spot” algorithms were used for three different ovarian structures: secondary follicles, antral follicles and corpora lutea. Then, the whole nervous network was reconstructed by “Cell” algorithm in “Surpass” panel. The data of each algorithm was extracted from “Statistics” in “Preferences” in “Edit” panel. In order to delete the data of neuron filaments, the biggest ganglion in whole image was isolated by “Crop 3D” in “Edit” panel and the area of this ganglia was examined by “Cell” algorithm. The areas higher than area of biggest ganglia were deleted, as well as their counts. The number and total area of neuronal filaments were calculated by subtracting the total number and area of whole image from the number and area of ganglia. For follicle and corpus luteum analysis, each follicle and its surrounding ganglia were isolated by “Crop 3D” in “Edit” panel. “Cell” algorithm was used for ganglia reconstruction in crops. Only two neuronal filaments were detected in these crop 3Ds and their data were manually deleted. The data of area and number of ganglia from each crop were extracted from “Statistics” in “Preferences” in “Edit” panel (Figure S2B).

### Data analysis

After data extraction from ImageJ and Imaris software, the data of area and number of structures (ganglia and neuronal filaments) obtained from image analysis were entered into Excel software. IBM SPSS Statistics 26 (SPSS for Windows, version 26, SPSS Inc., Chicago, Illinois, USA) software was used for statistical analysis. The mean differences between follicular groups were analyzed by one-way ANOVA and post hoc Tukey test. The correlation of the total ganglia area and number and the area of structures (follicles and corpora lutea) with each other in four different groups including total ovarian structures, secondary and antral follicles and corpora lutea were analyzed by Pearson correlation. All data were expressed as the mean ± standard error of the mean, and *p* value was considered less than 0.05 for statistical significance. GraphPad Prism (v7.0a, GraphPad Software, Inc., San Diego, CA, USA) software was used for drawing the graphs. The table format was “Grouped” and the data input was the mean of area and number of ganglia and standard error of the mean.

### Statement of ethics

All experimental protocols were approved by the Shiraz University Ethics Committee (project number: 97gcu4m148075). All methods were carried out in accordance with the World Medical Association Declaration of Helsinki. This study was carried out in compliance with the ARRIVE guidelines (http://www.nc3rs.org.uk/page.asp?id=1357). Efforts were made to minimize animal suffering and to reduce the number of animals used.

## Supplementary Information

Supplementary Information.

Supplementary Video.
