# Supplementary material for: Three-dimensional and two-dimensional relationships of gangliogenesis with folliculogenesis in mature mouse ovary: a Golgi–Cox staining approach
Source: Sci Rep. 2021 Mar 10;11:5547. doi: 10.1038/s41598-021-84835-0 (PMC7970916; doi:10.1038/s41598-021-84835-0)
Supplement: Supplementary file 1 — Supplementary Information. [file 41598_2021_84835_MOESM1_ESM.docx]

# **Three-dimensional and two-dimensional relationships of gangliogenesis with folliculogenesis in mature mouse ovary: A Golgi-Cox staining approach**

**Running title:** 3D imaging of gangliogenesis in ovary

Mohammad Ebrahim Asadi Zarch^a*^, Alireza Afshar^b*^, Farhad Rahmanifar^c*^, Mohammad Reza Jafarzadeh Shirazi^a†^, Mandana Baghban^d^, Mohammad Dadpasand^a^, Farzad Mohammad Rezazadeh^a^, Arezoo Khoradmehr^b^, Hossein Baharvand^e,f^, Amin Tamadon^b†^

^a^ Department of Animal Sciences, College of Agriculture, Shiraz University, Shiraz, Iran

^b^ The Persian Gulf Marine Biotechnology Research Center, The Persian Gulf Biomedical Sciences Research Institute, Bushehr University of Medical Sciences, Bushehr, Iran

^c^ Department of Basic Sciences, School of Veterinary Medicine, Shiraz University, Shiraz, Iran

^d^ Department of Obstetrics and Gynecology, School of Medicine, Shiraz University of Medical Sciences, Shiraz, Iran

^e^ Department of Stem Cells and Developmental Biology, Cell Science Research Center, Royan Institute for Stem Cell Biology and Technology, ACECR, Tehran, Iran

^f^ Department of Developmental Biology, University of Science and Culture, Tehran, Iran

† Correspondences:

Amin Tamadon, The Persian Gulf Marine Biotechnology Research Center, The Persian Gulf Biomedical Sciences Research Institute, Bushehr University of Medical Sciences, Bushehr, Iran; Postal Code: 75146-33196; Tel/fax: +98 77 3332 8724; Email: [amintamaddon@yahoo.com](mailto:amintamaddon@yahoo.com)

Mohammad Reza Jafarzadeh Shirazi, Department of Animal Sciences, College of Agriculture, Shiraz University, Shiraz, Iran; Postal Code: 71441-65186; Tel/fax: +98 71 3228 6073, e-mail: [jafarzd@shirazu.ac.ir](mailto:jafarzd@shirazu.ac.ir)

# **Supplementary Figures**


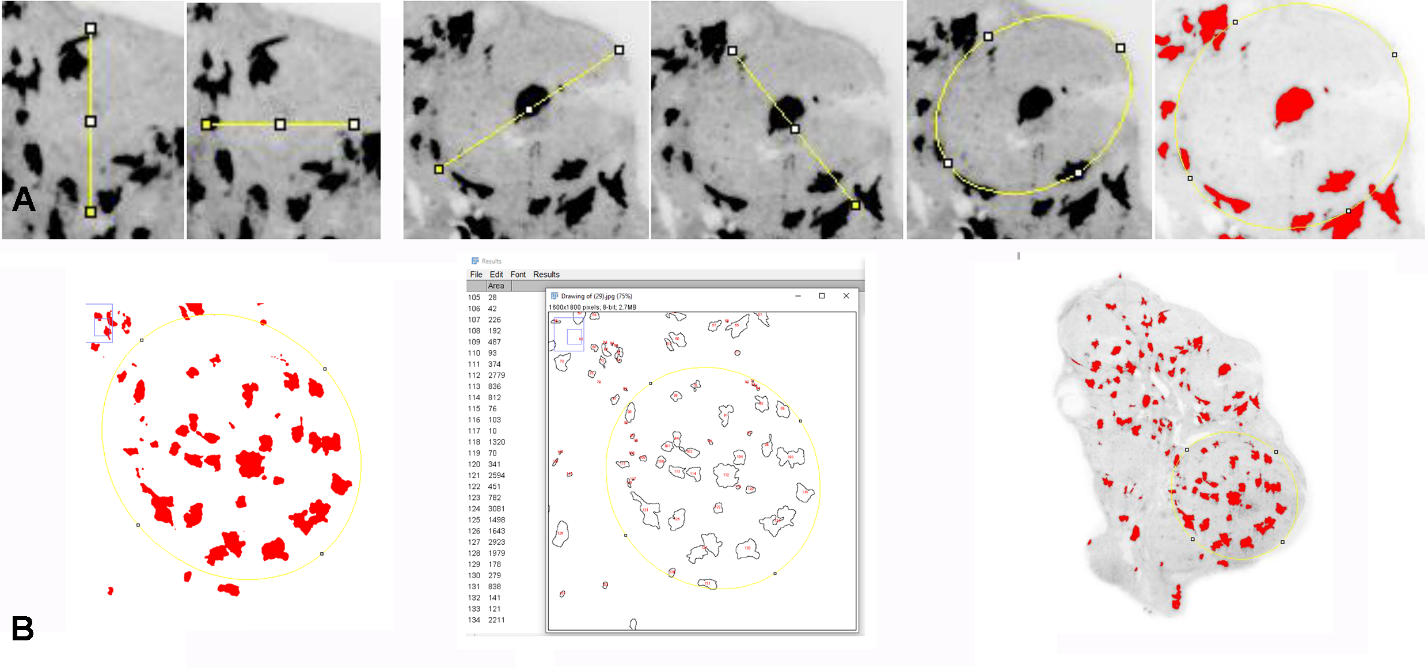


## Fig. S1. Two-dimensional analysis of ovarian structures and ganglia in Golgi Cox staining.

A) Steps of measuring the diameter and area of the ovarian follicle with “line” and “Oval” tools in ImageJ. B) Segmentation of ganglia in the ovary to count and measure areas of ganglia with ImageJ “threshold” and “analyze particle” algorithms.


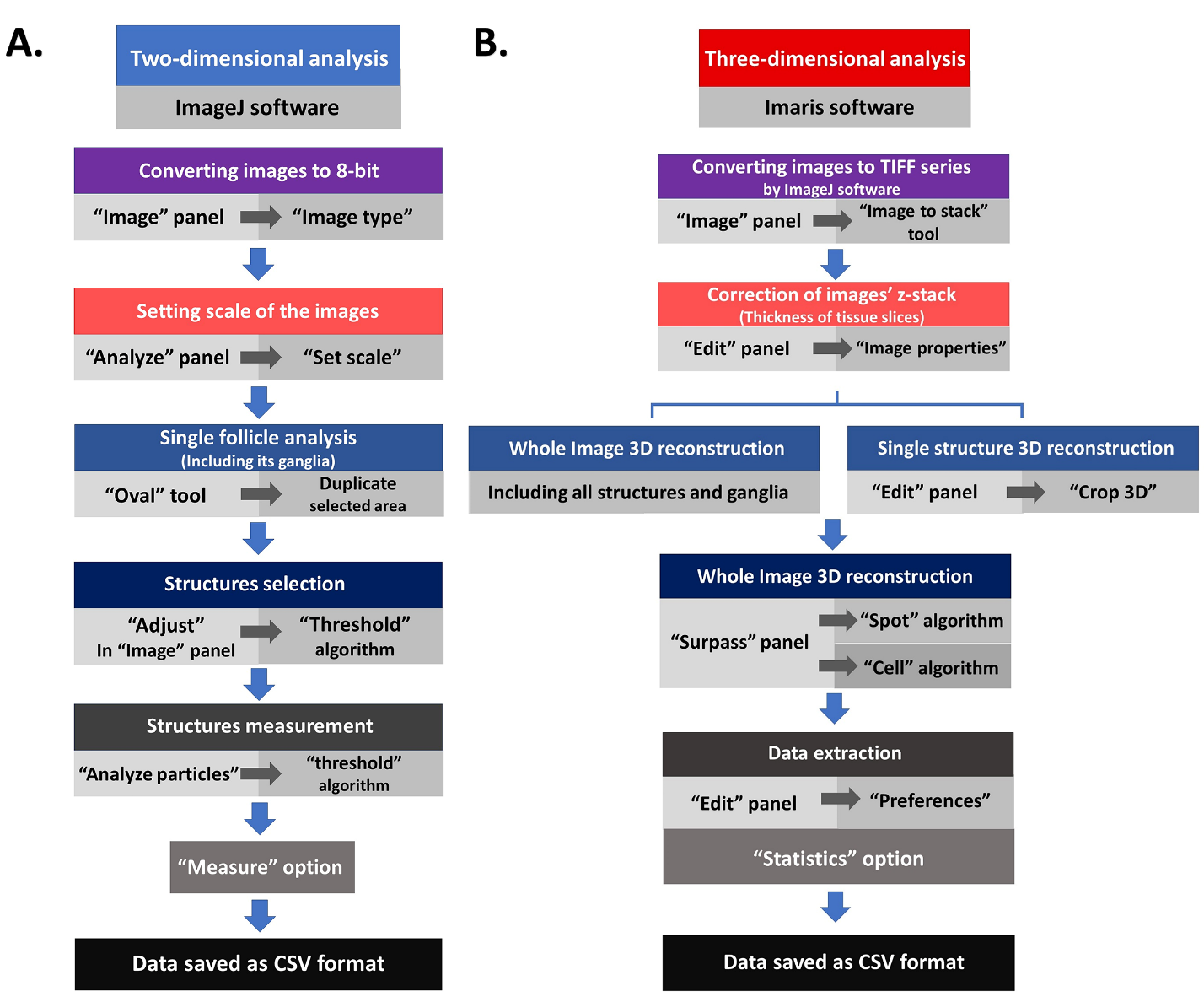


## Fig. S2. The flowcharts of the two-dimensional (2D) and three-dimensional (3D) analysis protocols. A) 2D analysis protocol. B) 3D analysis protocol.

# **Supplementary video**

## Video S1. 3D reconstruction of the ovarian structures and ganglia after Golgi Cox staining of ovary in diestrus mice.
